# Intraplaque hemorrhage is associated with higher structural stresses in human atherosclerotic plaques: an in vivo MRI-based 3d fluid-structure interaction study

**DOI:** 10.1186/1475-925X-9-86

**Published:** 2010-12-31

**Authors:** Xueying Huang, Zhongzhao Teng, Gador Canton, Marina Ferguson, Chun Yuan, Dalin Tang

**Affiliations:** 1 School of Mathematical Sciences, Xiamen University, Xiamen, Fujian 361005, PR China; 2 Mathematical Sciences Department, Worcester Polytechnic Institute, MA 01609, USA; 3 University Department of Radiology, University of Cambridge, Cambridge, UK; 4 Deparment of Radiology, University of Washington, Seattle, WA 98195, USA

## Abstract

**Background:**

Studies using medical images have shown that intraplaque hemorrhage may accelerate plaque progression and may produce a stimulus for atherosclerosis development by increasing lipid core and plaque volume and creating new destabilizing factors. Image-based 3D computational models with fluid-structure interactions (FSI) will be used to perform plaque mechanical analysis and investigate possible associations between intraplaque hemorrhage and both plaque wall stress (PWS) and flow shear stress (FSS).

**Methods:**

In vivo MRI data of carotid plaques from 5 patients with intraplaque hemorrhage confirmed by histology were acquired. 3D multi-component FSI models were constructed for each plaque to obtain mechanical stresses. Plaque Wall Stress (PWS) and Flow Shear Stress (FSS) were extracted from all nodal points on the lumen surface of each plaque for analysis.

**Results:**

The mean PWS value from all hemorrhage nodes of the 5 plaques combined was higher than that from non-hemorrhage nodes (75.6 versus 68.1 kPa, P = 0.0003). The mean PWS values from hemorrhage nodes for each of the 5 plaques were all significantly higher (5 out of 5) than those from non-hemorrhage nodes (P < 0.05). The mean FSS value from all hemorrhage nodes of the 5 plaques combined was 30.4% higher than that from all non-hemorrhage nodes (15.0 versus 11.5 dyn/cm^2^, P = 0.0002). However, the mean flow shear stress values from individual cases showed mixed results: only one out of five plaques showed mean FSS value from hemorrhage nodes was higher than that from non-hemorrhage nodes; three out of five plaques showed that their mean FSS values from hemorrhage nodes were lower than those from non-hemorrhage nodes; and one plaque showed that the difference had no statistical significance.

**Conclusion:**

The results of this study suggested that intraplaque hemorrhage nodes were associated with higher plaque wall stresses. Compared to flow shear stress, plaque wall stress has a better correlation with plaque component feature (hemorrhage) linked to plaque progression and vulnerability. With further validation, plaque stress analysis may provide additional stress indicators for image-based vulnerability assessment.

## Introduction

Atherosclerotic plaque rupture is the primary cause of acute cardiovascular syndromes, such as heart attack and stroke, and may occur without any warning [1-3]. Vulnerable plaques often have a large necrotic core covered by a thin fibrous cap and may contain intraplaque hemorrhage, calcification, and inflammation [3-5]. Currently, interventional diagnosis is still mainly based on the degree of luminal stenosis/plaque severity as measured by angiography (X-ray, MRI, ultrasound, or computed tomography). However, there is growing evidence suggesting that plaque severity alone is insufficient for identifying the critical condition [6].

From a mechanical point of view, plaque rupture is likely to occur when the mechanical stress exceeds the material strength of fibrous cap. Therefore, it has been hypothesized that critical stress conditions in the plaque may be closely related to plaque rupture and can be combined with current image-based assessment techniques for more accurate plaque evaluation and vulnerability assessment [7,8]. Computational models combing mechanical factors and morphologic information have been introduced by several groups to perform plaque mechanical analysis and identify additional critical mechanical indicators to improve the current histology and image-based plaque assessment [9-25]. For structure-only models, Holzapfel et al. introduced multi-layer anisotropic plaque models and showed that stress predictions from their models varied from single-layer isotropic models by 50%-200% or more [9,10]. Li et al. performed 2D structural analysis based on in vivo MRI of carotid arteries and found that wall stress was higher in ruptured plaques than in non-rupture plaques [14]. Tang et al. introduced the first multi-component FSI plaque model which integrates plaque morphology, composition, fluid and structural forces together to provide more complete mechanical stress analysis for vulnerable plaques, compared to fluid- or solid-only models [11]. For the impact of specific plaque characteristics and components, Bluestein et al. investigated the effect of microcalcifications on vulnerable plaque mechanics using FSI modeling [12]. Loree et al. studied effects of fibrous cap thickness and their results indicated that reducing fibrous cap thickness dramatically increased peak circumferential stress in the plaque [15]. Sadat et al. investigate the impact of plaque hemorrhage and its age on structural stresses in atherosclerotic plaques using biomechanical stress simulations [16]. Due to the importance of flow shear stress and artery geometrical parameters, using flow-only models, Steinman et al. and Lee at al. investigated influence of complex vessel geometry on flow behaviors using realistic plaque geometries [17,18]. For coronary arteries, Zhu et al. also analyzed geometrical parameters of human coronary arteries for the potential of predicting coronary artery diseases [19]. The influence of curvature dynamics and cyclic bending on coronary plaque stress and flow behaviors was investigated by Prosi et al. and Tang et al. [20,21]. Suo et al. studied blood flow patterns in the proximal human coronary arteries and reported that low wall shear stress was co-located with increased incidence of lesions, and higher wall shear stresses were associated with lesion-resistant areas [22]. Groen and Wentzel et al. reported a follow-up case study showing high flow shear stress region was associated with site of plaque rupture [23]. Tang et al. used image-based FSI models to quantify critical mechanical conditions which may be related to plaque rupture and progression [7,8,11,24-27]. In particular, they indicated that global maximum plaque stress often occur at healthy sites and that it is the critical plaque stress at vulnerable sites that may be more closely linked to plaque rupture [7,8]. In a more recent paper using FSI models based on in vivo MRI of human carotid plaques with and without rupture, they provided some initial evidence that higher plaque wall stress were associated with plaque rupture [24]. Recent reviews of image-based computational modeling effort can be found in [24,28]

Growing evidence suggests that intraplaque hemorrhage is related to rapid plaque progression and rupture may produce a stimulus for the atherosclerosis by increasing lipid core and plaque volume and creating new destabilizing factors [29-31]. Intraplaque hemorrhage may contribute to the deposition of free cholesterol and macrophage infiltration. Erythrocyte membranes from intraplaque hemorrhage into the necrotic core are a source of free cholesterol and may become a driving force in the progression of atherosclerosis. Multi-contrast MRI techniques have been shown to be able to non-invasively characterize carotid intraplaque hemorrhage [32].

To date, the impact of intraplaque hemorrhage on plaque wall stress (PWS) and flow shear stress (FSS) has not been analyzed considering the coupling of fluid and solid mechanics forces. The goal of this study is to determine the potential association between locations of intraplaque hemorrhage and mechanical plaque wall stress and flow shear stress by building fluid-structure interaction (FSI) models of carotid atherosclerotic plaques containing intraplaque hemorrhage. 3D FSI models were constructed based on in vivo MRI data acquired from 5 patients where intraplaque hemorrhage was detected and validated with histological images.

## Methods

### MRI Data Acquisition

3D in vivo MR images of human atherosclerotic carotid plaques with hemorrhages were acquired from 5 patients scheduled for carotid endarterectomy (age: 57 to 82, mean = 67, all male) by the Vascular Imaging Laboratory (VIL) of the University of Washington (UW) using protocols approved by UW Institutional Review Board with patient consent obtained. Details of MRI acquisition protocols were previously reported [24,33-35]. All images were obtained with the following parameters: 16 × 16 cm^2 ^field-of-view, 256 × 256 matrix size, and 2 mm slice thickness. After interpolation, the in-plane resolution was 0.31 × 0.31 mm^2^. The longitudinal coverage covered the bulk region of each lesion.

**Figure 1:**
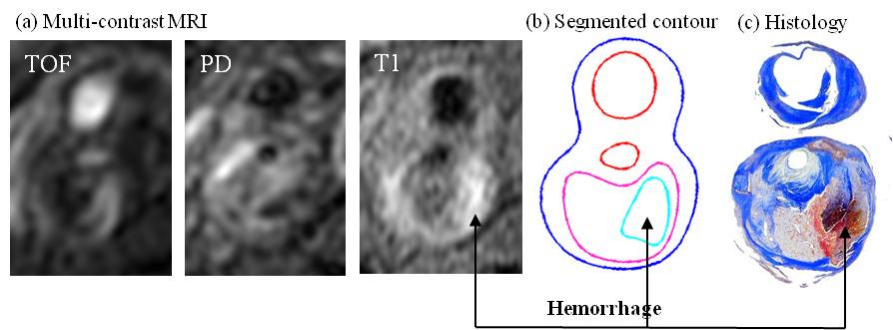
**TOF-, PD-, and T1-weighted MR images of a human carotid plaque sample with hemorrhage validated by histology**.

**Figure 2:**
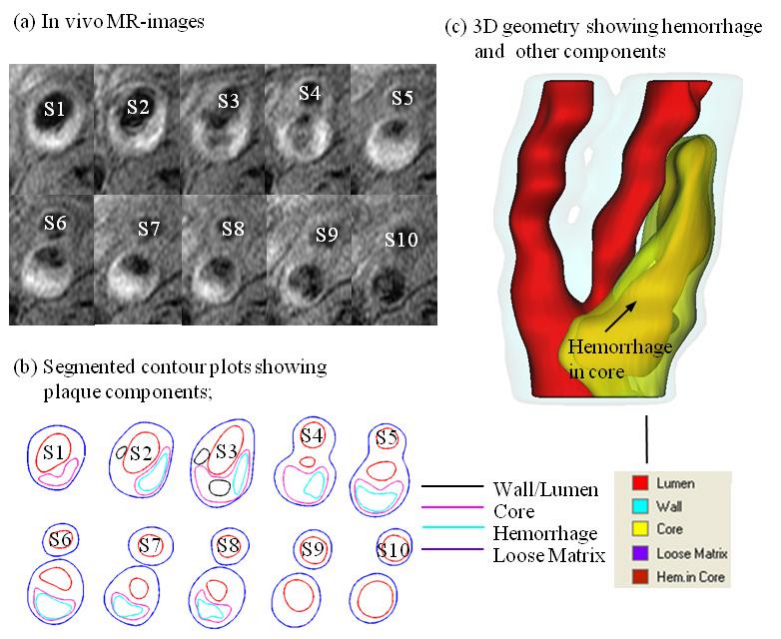
**T1-weighted MR images and segmented contour plots showing Hemorrhage**. (a) In vivo MR-images; (b) Segmented contour plots showing plaque components; (c) 3D geometry showing hemorrhage and other components.

MR images were segmented using custom-made analysis tools (CASCADE developed at the Vascular Imaging Laboratory at University of Washington, and APIA, from Washington University) to identify lipid-rich necrotic core (LRNC), loose matrix (LM), calcification (Ca), thrombus, and intraplaque hemorrhage[32-37]. Figure 1 shows a human atherosclerotic carotid plaque slice (S4) with hemorrhage validated by histology. Figure 2 gives a set of 10 slices of T1-weighted MR images and segmented contour plots.

### FSI Computational Model

Both the artery wall and plaque components were assumed to be hyperelastic, isotropic, incompressible and homogeneous. Blood flow was assumed to be laminar, Newtonian, viscous and incompressible. The unsteady incompressible Navier-Stokes equations with arbitrary Lagrangian-Eulerian (ALE) formulation were used as the governing equations. A no-slip condition between all interfaces was assumed. Patient-specific systolic and diastolic pressure conditions from the last hospital admission were used as the maximum and minimum of a typical arterial pressure waveform at the inlet of the artery. Pressure waveforms at the outlets were adjusted to obtain flow rates within physiological range. The modified Mooney-Rivlin model was used to describe the material property of each component in the plaque [11,24,38],(1)

Where  are the first and second strain invariants, ***C ***=[*C*_*ij*_] = **X**^^T^^**X **is the right Cauchy-Green deformation tensor, **X**=[X_ij_] = [∂x_i_/∂a_j_], {x_i_} is current position, {a_i_} is original position, *c*_*i *_and *D*_*i *_are material parameters chosen to match experimental measurement [26,27]. In this paper, the following parameter values were chosen: vessel/fibrous cap, *c*_1 _= 36.8 KPa, *D*_*1 *_= 14.4 KPa, *D*_*2 *_= 2; calcification, *c*_*1 *_= 368 KPa, *D*_*1 *_= 144 KPa, *D*_*2 *_= 2.0; lipid core/hemorrhage/ulcers, *c*_*1 *_= 2 KPa, *D*_*1 *_= 2 KPa, *D*_*2 *_= 1.5; loose matrix, *c*_*1 *_= 18.4 KPa, *D*_*1 *_= 7.2 kPa; *D*_*2 *_= 1.5. *c*_*2 *_= 0 was set for all materials [16,26,27].

### 3D Re-Construction, Shrink-Stretch Process, and Mesh Generation Process

Under the in vivo condition, the arteries are axially stretched and pressurized. Therefore, axial and circumferential shrinking should be applied to generate the no-load starting shape for the computational simulations. The shrinkage in the axial direction was 9% so that the vessel would regain its in vivo length with a 10% axial stretch. Circumferential shrinkage for lumen and outer wall was determined so that: 1) total mass volume was conserved; and 2) plaque geometry after 10% axial stretch and pressurization had the best match with the original in vivo geometry [39]. Because advanced plaques have complex irregular geometries with component inclusions that are challenging for mesh generation, a component-fitting mesh generation technique was developed to generate mesh for these models. Using this technique, the 3D plaque domain was divided into hundreds of small "volumes" to curve-fit the irregular plaque geometry with plaque component inclusions [21]. Since hemorrhages usually occur within the lipid rich necrotic core with very irregular shape, they were treated as the same as lipid to reduce the model construction effort. The error caused by this simplification will be discussed in Section 4. 3D surfaces, volumes and mesh were created with ADINA (ADINA R & D, Inc., Watertown, MA, USA). Each plaque model required about 3600 small volumes to be created to fit the shape of wall and components, and about 1000 small volumes for the corresponding fluid domain.

### Solution Method

The coupled FSI plaque models were solved by a commercial finite-element package ADINA. This software uses unstructured finite element methods for both fluid and solid models. Nonlinear incremental iterative procedures were used to handle fluid-structure interactions. The governing finite element equations for both the solid and fluid models were solved by the Newton-Raphson iteration method. Details of the models and methods are given in Tang et al. [11,24] and Bathe [38].

### Plaque Stress/Strain and Flow Shear Stress Data Extraction

**Figure 3:**
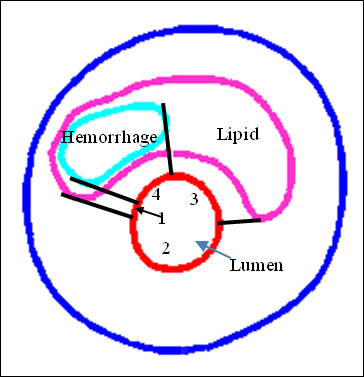
**Schematic drawing demonstrating the node-type assignment method for nodal point on lumen surfaces**. Node type was defined as the closest component the node was covering, or hemorrhage if it was inside the lipid. Zones 1 & 3 are lipid zones; Zone 2 is a normal wall zone; Zone 4 is a hemorrhage zone.

Data for PWS, and FSS were extracted from 3D FSI solutions for all integral nodes (total: structure: 3245; fluid: 2828) on lumen surfaces of 5 plaque models for analysis. For each nodal point, plaque maximum principal stress was used for PWS and maximum flow shear stress was used for FSS. Each node was assigned a node type (hemorrhage, lipid, calcification, and wall if the node was not covering any component) according to its location and the component it was covering. Figure 3 gives a schematic drawing demonstrating the node-type assignment method for nodal point on lumen surfaces. Node type for each node was defined as the closest component the node was covering, or hemorrhage if it was inside the lipid as shown in Figure 3.

### Statistical Analysis

Mean values of PWS and FSS for each node type were calculated for analysis and comparisons. A *t*-test was used for statistical analysis to compare PWS and FSS from the hemorrhage and non-hemorrhage nodes of each group. A *P*-value of less than 0.05 was deemed a significant difference.

## Results

**Figure 4:**
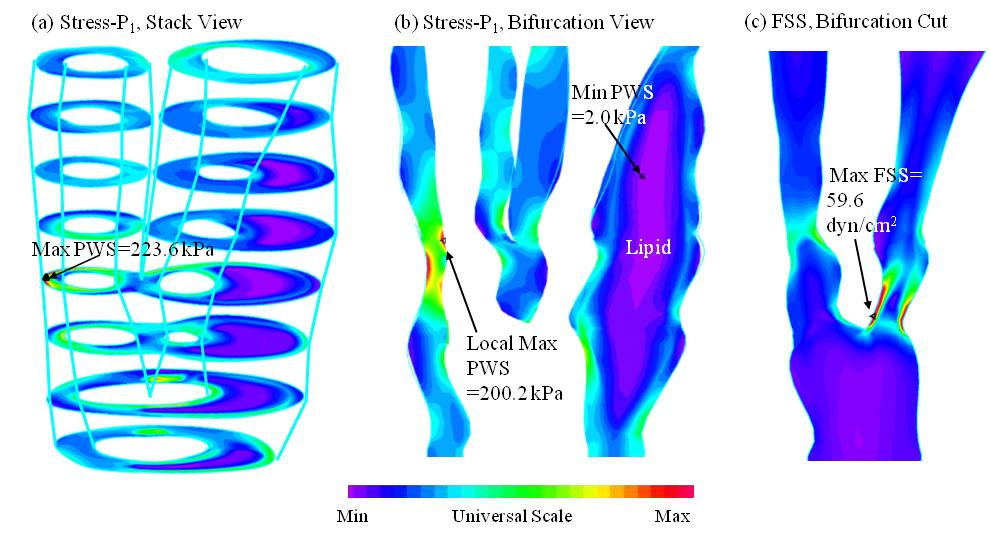
**Band plots of Plaque Wall Stress (Stress-P**_**1**_**) and Flow Shear Stress (FSS) for the plaque sample given in Fig 1**. (a) a stack view of PWS; (b) PWS plot on a longitudinal cut showing the bifurcation; (c) FSS plot on a longitudinal cut.

Figure 4 gives an overview of solution features from 3D FSI models using the plaque sample given in Figure 2. Maximum principal stress (Stress-P_1_) and flow maximum shear stress (FSS) on stacked cross-section slices, and a bifurcation cut (B-cut) surface were presented. Maximum Stress-P_1 _was observed at the node which was a healthy site due to the curvature. To analyze mechanical conditions (structural stress and flow shear stress) on the lumen surface corresponding to different tissue types and to identify differences between hemorrhage and non-hemorrhage nodes, data were extracted from the full 3D FSI solutions and mean values of PWS and FMSS of hemorrhage (Hemo), lipid core, calcification (Ca), wall, and all non-hemorrhage nodes are summarized in Tables 1 and 2. kPa and dyn/cm^2 ^are used as the units for PWS and FSS, respectively (1 kPa = 10^4 ^dyn/cm^2^).

**Table 1:** Summary of mean plaque Stress-P_1 _values of hemorrhage, lipid core, calcification, wall, all nodes, and all non-hemorrhage nodes for the 5 plaque samples.

Case	Hemo		Wall		Lipid		Ca		All Nodes		All Non-Hemo		P-value
		
	mean	n	mean	n	mean	n	mean	n	mean	n	mean	n	
P1	67.8	87	55.7	317	77.3	93	53.4	11	61.7	508	60.4	421	0.0197
P2	87.6	24	62.1	278	59.1	164			62.4	466	61.0	442	0.0204
P3	109.4	5	74.5	357	77.7	110	45.2	15	74.8	487	74.4	482	<0.0001
P4	82.7	75	72.4	416	54.9	111	30.4	23	69.5	650	67.1	550	<0.0001
P5	82.1	47	71.9	467	95.3	46			74.7	560	74.0	513	0.0473
All 5 p	75.6	238	68.2	1835	68.5	524	40.1	49	68.8	2671	68.1	2433	0.0003

The p-values are for the Hemorrhage vs. non-Hemorrhage. Unit: KPa.

### Hemorrhage Nodes Were Associated With Higher PWS Values Compared to Non-Hemorrhage Nodes

Table 1 shows that the mean PWS values from hemorrhage nodes of these 5 cases were (unit: kPa) 67.8, 87.6, 109.4, 82.7, and 82.1, respectively, with an average of 85.9 ± 15.1; the mean PWS values from non-hemorrhage nodes of these 5 cases were 60.4, 61.0, 74.4, 67.1, 74.0, and 66.1, respectively, with an average = 67.4 ± 6.8. The mean PWS values from hemorrhage nodes for each of the 5 plaques were all significantly higher (5 out of 5) than those from non-hemorrhage nodes (P < 0.05). The mean PWS value from all hemorrhage nodes combined from all the 5 plaques was 75.6 kPa, which is 11.01% higher than the mean PWS value (68.1 kPa) from all non-hemorrhage nodes combined from the 5 plaques (P = 0.0003).

### Overall Results Showing Hemorrhage Nodes Were Associated With Higher FSS Values Compared to Non-Hemorrhage Nodes, but Individual Cases Differ

Table 2 indicates that mean FSS from all hemorrhage nodes combined from the 5 plaques (15.0 dyn/cm^2^) was 30.4% higher than that (11.5 dyn/cm^2^) from all non-hemorrhage nodes (P = 0.0002). However, mean FSS values for each individual plaque showed mixed results. Of the 5 cases, one plaque showed mean FSS value of hemorrhage nodes was higher than that from non-hemorrhage nodes (23.7 versus 10.0, P < 0.0001); three plaques showed that mean FSS values from hemorrhage nodes (17.6, 11.2, 7.1) were less than mean FSS values from non-hemorrhage nodes (20.8, 15.2, 11.7), P < 0.05; and anther plaque showed mean FSS (14.3) was greater than that (12.4) from non-hemorrhage nodes, but the difference was not statistically significant (P = 0.3223).

**Table 2:** Summary of mean Fluid shear stress values of hemorrhage, lipid core, calcification, wall, all nodes, and all non- hemorrhage nodes for the 5 plaque samples.

Case	Hemo		Wall		Lipid		Ca		All Nodes		All Non-Hemo		P-value
		
	mean	n	mean	n	mean	n	mean	n	mean	n	mean	n	
P1	14.3	87	10.8	334	18.4	93	7.5	11	12.7	525	12.4	438	0.3223
P2	17.6	24	20.4	291	21.4	164			20.6	479	20.8	455	0.0103
P3	11.2	4	11.7	271	25.1	102	7.8	8	15.1	385	15.2	381	0.0014
P4	23.7	61	10.9	275	8.1	61	1.0	16	12.0	413	10.0	352	<0.0001
P5	7.1	44	12.2	477	7.2	49			11.4	570	11.7	526	<0.0001
All 5 P	15.0	212	10.9	1577	14.3	420	4.6	35	11.8	2244	11.5	2032	0.0025

The p-values are for the Hemorrhage vs. non-Hemorrhage comparisons. Unit: dyn/cm^2^.

## Discussion

### Structural Stress Was Associated with Intraplaque Hemorrhage and May Be an Indicator for Plaque Vulnerability Assessment

To our knowledge, this is the first 3D Fluid-Structure Interaction study that attempts to quantify the differences of atherosclerotic structural stress and flow behaviors between hemorrhage and non-hemorrhage locations with multiple in vivo patient data. In all five cases, the mean PWS values of hemorrhage nodes are higher than other non-hemorrhage nodes (10-50% higher, all statistically significant). Since intraplaque hemorrhage has been reported to be involved in plaque progression and can be considered as a destabilizing factor of atherosclerosis plaques [13,29,40-42], analyzing the association between intraplaque hemorrhage and biomechanical stresses might improve the understanding of the role of the mechanical forces in the disease. The results obtained provided some initial evidence that high plaque stresses are linked with plaque vulnerability. In vivo MRI-based computational simulations integrate the information of plaque morphology, material properties of the plaque components, and local flow patterns. The structural stress might have the potential to be an indicator for plaque vulnerability assessment in addition to plaque morphology assessment. However, even with the indication that 3D structural stress is associated with plaque vulnerability, large-scale longitudinal patient studies are still needed to further validate our findings.

### Association of Flow Shear Stress with Plaque Rupture

There has been great interest and a considerable effort spent investigating the relationship between FSS and the progression and rupture of atherosclerotic plaque [43,44]. The overall results from this study support the hypothesis that a high flow shear stress region is associated with plaque vulnerability. However, the individual results indicated that only 1 out of 5 plaques show FSS from hemorrhage nodes higher than that from non-hemorrhage nodes. Larger-scale studies are needed to further clarify the role of FSS in plaque rupture progress. Keeping in mind that the mean value of FSS is 15.0 dyn/cm^2^, which is only 0.00198% of the mean value of PWS (75.6 kPa), contributions from flow shear stress acting as a rupture trigger may be much smaller than that from structural stress. However, high flow shear stress on the lumen surface may have long-term effects leading to endothelial dysfunction and lumen surface weakening.

### Limitations

**Figure 5:**
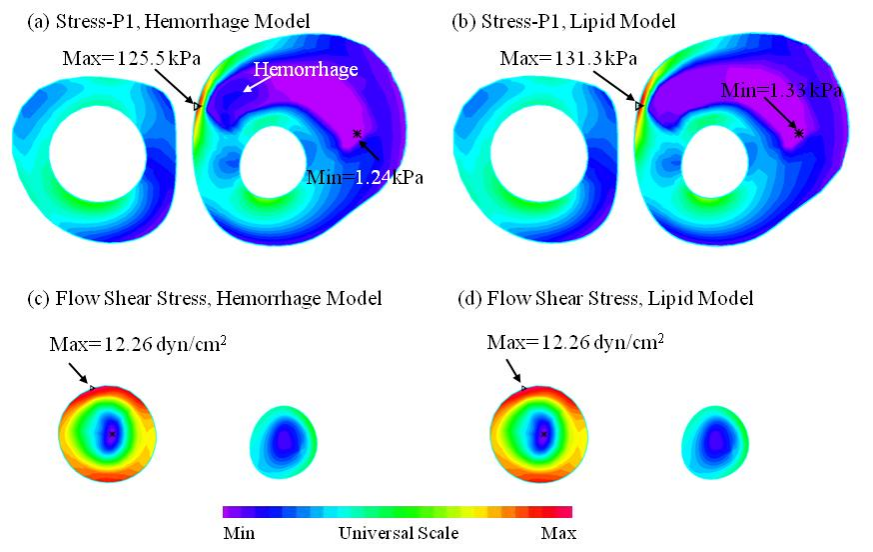
**Comparison of PWS on a z-cut surface (Slice 8) from the model with hemorrhage and the model where hemorrhage replaced by Lipid: (a) PWS on Z-cut, hemorrhage model;(b) PWS on Z-cut, Lipid model; (c) FSS on Z-cut, hemorrhage model;(d) FSS on Z-cut, Lipid model**.

In this paper, we treated intraplaque hemorrhages (which were contained in lipid cores) the same as lipid to reduce model construction effort. To justify this modeling approach, two models (called the lipid model and hemorrhage model, respectively) were constructed for one plaque sample with and without the replacement simplification. Figure 5 shows the comparison of PWS and FSS distributions obtained from the two models using one cut surface showing the hemorrhage and lipid components. FSS distributions obtained from these two models were almost identical on the cut surface. The mean FSS value from the hemorrhage nodes was 17.56 ± 4.43 dyn/cm^2 ^for the hemorrhage model, which was almost the same as that from the lipid model (17.24 ± 4.36 dyn/cm^2^). The result is reasonable because the lipid model did not change the fluid domain much. The PWS distributions from the two models were slightly different in the hemorrhage region, as expected. However, the mean PWS value from hemorrhage nodes was 86.5 ± 49.9 kPa, which was 2.19% less than that from lipid model (88.4 ± 49.3 kPa). This is because the material property of hemorrhage is similar to that of lipid used in this study. The error for both PWS and FSS are less than 3%. Therefore, the lipid model provided reasonable FSS and PWS results for our analysis.

It should be emphasized that the sample size (n = 5) is really too small to draw any strong conclusions. This is especially true for the FSS results. Looking at Table 2, P4 data pushed the average FSS of hemorrhage from the 5 samples significantly larger than that of all non-hemorrhage components (15 vs. 11.5, P = 0.0025). Combining that with the fact that 3 out of the 5 cases had mean FSS from hemorrhage nodes lower than those from non- hemorrhage nodes and one case with no significant difference, we should say that our results for the role of FSS was mixed.

Other model limitations include: a) Patient-specific material properties were not included due to the inability to measure these material properties using current techniques; b) arm systole and diastole pressures taken at the past scan visit were used to scale the pressure profile used in the simulations since pressure conditions at the location of the plaque were not available; c) lumen surface inflammation and erosion were not considered in this study since current in vivo MRI technology could not accurately provide these data.

## Conclusion

The associations between intraplaque hemorrhage and plaque wall stress and flow shear stress were investigated using 3D in vivo MRI-based FSI models. All 5 cases studied showed that intraplaque hemorrhage nodes were associated with higher plaque wall stress (10-50% higher by mean value) compared with non-hemorrhage nodes. The association of flow shear stress with hemorrhage showed mixed results when the cases were considered combined and individually. The close association between plaque wall stress and intraplaque hemorrhage indicates that plaque wall stress may be useful in plaque vulnerability assessment. Large-scale studies are needed to further validate our findings.

## Competing interests

Other than the grants listed in the acknowledgement section, the authors declare that they have no other competing interest.

## Authors' contributions

DT, XH, ZT and CY (Yang) were responsible for computational modeling and data analysis part. CY Yuan), GC, and MF were responsible for the MRI data and histology data acquisition and the segmentation part. All authors 1) have made substantial contributions to conception and design, or acquisition of data, or analysis and interpretation of data; 2) have been involved in drafting the manuscript or revising it critically for important intellectual content; and 3) have given final approval of the version to be published. Each author has participated sufficiently in the work to take public responsibility for appropriate portions of the content.

## Authors' information

Tang's group has been publishing image-based modeling work in recent years. For more information, please visit Tang's website: http://users.wpi.edu/~dtang/.

Dr. Yuan's group and their lab (Vascular Imaging Laboratory, University of Washington) have been developing MR imaging methods and have published extensively in this area. For more information, please visit their website: http://www.rad.washington.edu/research/our-research/groups/vil.
